# Supplementary material for: Repression of GhTUBB1 Reduces Plant Height in Gossypium hirsutum
Source: Int J Mol Sci. 2023 Oct 21;24(20):15424. doi: 10.3390/ijms242015424 (PMC10607470; doi:10.3390/ijms242015424)
Supplement: Supplementary file 1 [file ijms-24-15424-s001.zip › Supplementary Figures.pdf]

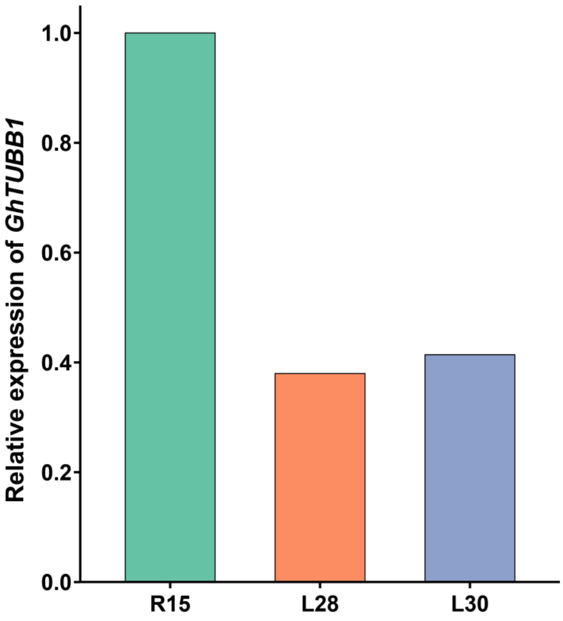


Figure S1. Relative expression of GhTUBB1 determined using previously published RNA sequencing data.

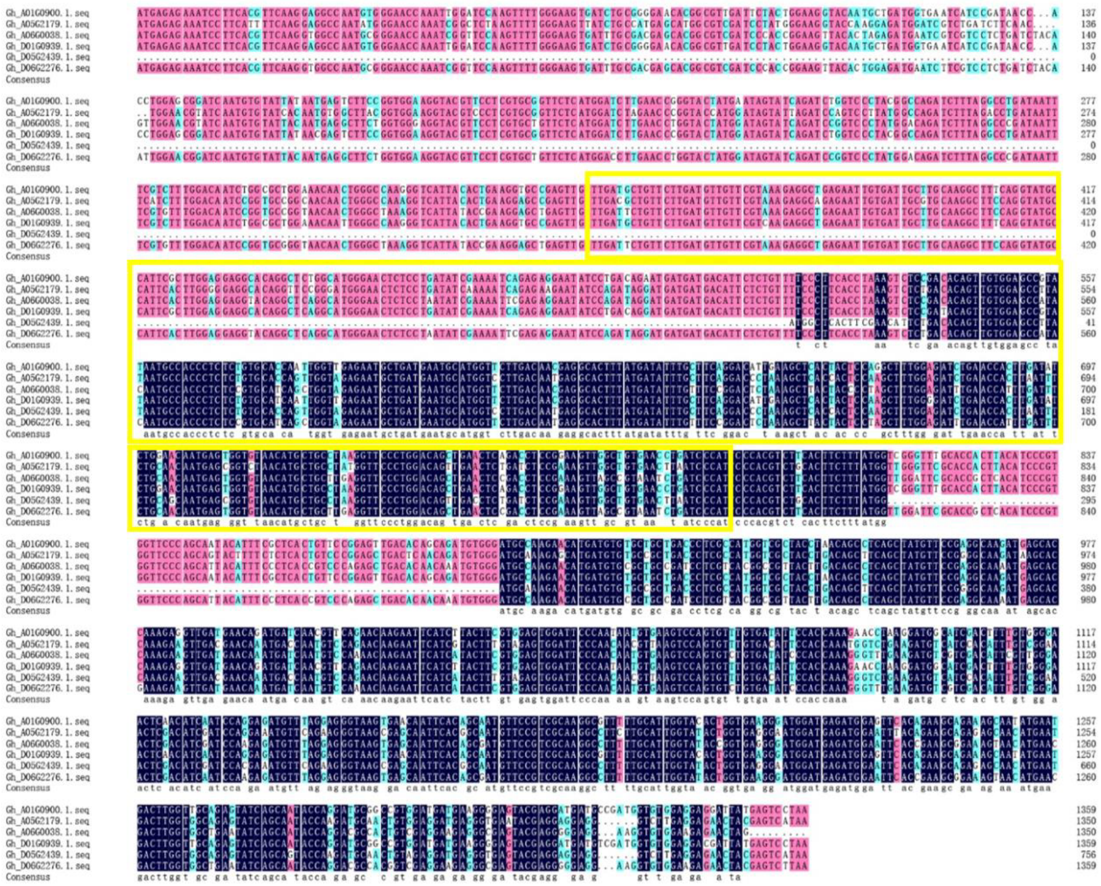

Figure S2. Alignment of six *Gossypium hirsutum GhTUBB*1 gene sequences. The yellow frames indicate regions that were targeted by VIGS.
